# Comparative Performance Assessment of Fiber Bragg Grating Sensors and PCB Accelerometers for Field-Based Dynamic Characterization of Stay Cables

**DOI:** 10.3390/s26144437

**Published:** 2026-07-13

**Authors:** Do Hong Phuc, Ly Hoang Mai, Phi Van Toan, Lien Thi Ngoc Truong, Le Van Vu, Nguyen Thi Cam Nhung

**Affiliations:** 1 Vietnam-Korea Institute of Science and Technology, Hoa Lac High Tech Park, Hanoi 10000, Vietnam; dhphuc@mst.gov.vn (D.H.P.); lhmai@mst.gov.vn (L.H.M.); ttnlien@mst.gov.vn (L.T.N.T.); 2 School of Materials Science and Engineering, Hanoi University of Science and Technology, Hanoi 10000, Vietnam; toan.phivan@hust.edu.vn; 3 Faculty of Civil Engineering, University of Transport and Communications, Hanoi 10000, Vietnam; vulv2704@gmail.com

**Keywords:** stay cable, Fiber Bragg Grating sensor, PCB accelerometer, dynamic characterization, vibration monitoring, modal identification, field measurement

## Abstract

Stay cables are critical components of cable-stayed bridges, and their dynamic characteristics provide essential information for vibration assessment and structural health monitoring. This study presents a comparative performance assessment of Fiber Bragg Grating (FBG) sensors and PCB accelerometers for field-based dynamic characterization of stay cables. Field measurements were conducted on selected stay cables of My Thuan Bridge under normal operating conditions. Since PCB accelerometers measure acceleration, whereas FBG sensors capture dynamic strain or wavelength-shift responses, the comparison was performed primarily in the frequency domain rather than through direct time-domain amplitude equivalence. The measured responses were processed using signal preprocessing, power spectral density analysis, and covariance-driven stochastic subspace identification. Dominant dynamic frequencies were identified from both sensing systems and compared using frequency agreement, frequency deviation, spectral peak consistency, and practical field applicability. The results show that the common frequencies identified from FBG and PCB measurements are in good agreement, with an overall mean relative difference of 0.94%. The FBG sensors also detected additional candidate frequency components that showed an approximately regular progression consistent with stay-cable vibration, although these components require further validation. These findings indicate that FBG sensing can provide reliable and complementary frequency-domain information for stay-cable dynamic characterization. The study demonstrates the feasibility of using FBG sensors as an alternative or complementary sensing technique to conventional accelerometers for field-based vibration monitoring of cable-stayed bridges.

## 1. Introduction

Structural Health Monitoring (SHM) has become an important approach for ensuring the safety, serviceability, and long-term performance of civil infrastructure systems [1,2]. Bridges are continuously subjected to traffic loads, wind actions, temperature variations, material deterioration, fatigue effects, and other environmental factors [3]. These factors may gradually change structural behavior and reduce the reliability of bridge components. Therefore, inspections must be performed regularly for bridge maintenance [4]. Traditional inspections are typically conducted at discrete intervals and often rely on visual observations, so they may not provide continuous, quantitative information on the actual structural condition. For large-scale bridges or difficult-to-access structural components, these methods of inspections may be difficult, time-consuming, or insufficient for continuous condition assessment [5].

To overcome these limitations, SHM systems have been increasingly developed and implemented in bridge engineering [6]. SHM system integrates sensing devices, data-acquisition units, signal-processing techniques, and structural assessment methods [7,8]. Depending on the monitoring objective, SHM may involve measuring strain, displacement, acceleration, temperature, inclination, vibration, or other response quantities [9,10]. SHM enables engineers to evaluate structural performance, identify abnormal changes, and support maintenance decision-making [11]. Among different SHM approaches, vibration-based monitoring has received significant attention [12]. The dynamic characteristics of a structure are closely related to its mass distribution, stiffness, damping, and boundary conditions. Natural frequencies, damping ratios, mode shapes, and spectral response features are commonly used to characterize structural dynamic behavior. Vibration-based information can provide insight into both global and local structural responses under ambient excitation, traffic loading, wind action, and other operational sources [13]. Vibration-based SHM has become a useful tool for assessing bridge performance and characterizing dynamic behavior [14,15].

Cable-stayed bridges represent an important class of long-span bridge structures for which SHM is particularly valuable [16]. In these bridges, stay cables play a key role in transferring loads from the bridge deck to the pylons and maintaining the overall structural configuration [17,18]. Due to their slenderness, flexibility, low inherent damping, and direct exposure to environmental actions, stay cables are highly sensitive to dynamic excitation [19]. Cable vibrations may be induced by wind, rain–wind interaction, traffic-induced bridge motion, deck–cable interaction, vortex shedding, and other aerodynamic or operational effects [20]. Excessive or persistent cable vibration may affect serviceability, accelerate fatigue damage in cable anchorage regions, and reduce the durability of cable protection systems. Therefore, reliable measurement and evaluation of stay-cable dynamic characteristics are important for the long-term monitoring of cable-stayed bridges.

The dynamic characteristics of stay cables, such as dominant natural frequencies, spectral peak distribution, damping behavior, and response stability, provide essential information for understanding their vibration behavior [21]. In field monitoring applications, these characteristics are often identified from measured vibration responses using time-domain and frequency-domain signal processing techniques. Frequency-domain analysis is commonly adopted because the dominant modal frequencies of stay cables can usually be observed as clear peaks in the response spectra [22,23]. For this reason, accurate and reliable sensing systems are crucial for obtaining high-quality vibration data and ensuring the robustness of dynamic identification results [24].

Conventional piezoelectric accelerometers, including PCB accelerometers, have been widely used for stay-cable vibration measurement and experimental modal analysis [25,26]. These sensors provide direct acceleration responses with high sensitivity, broad frequency range, and mature data acquisition procedures. Acceleration data can be conveniently processed using methods such as Fast Fourier Transform, power spectral density estimation, frequency-domain decomposition, and operational modal analysis [27]. Because of these advantages, accelerometers are often considered reliable reference sensors for field vibration testing. However, accelerometer-based measurements may require careful mounting, electrical wiring, sensor protection, and access to the cable surface [28]. These requirements may increase the complexity of field deployment, especially when long-term monitoring or multi-point measurement is needed [29].

In recent years, FBG sensing technology has emerged as a promising alternative for structural monitoring applications [30,31]. FBG sensors measure changes in reflected wavelength caused by strain and temperature variations in the optical fiber. Compared with conventional electrical sensors, FBG sensors offer several advantages, including compact size, lightweight configuration, immunity to electromagnetic interference, corrosion resistance, multiplexing capability, and suitability for long-distance signal transmission [32,33]. These features make FBG sensors attractive for bridge SHM, particularly in applications requiring long-term monitoring or multiple sensing points over large structural systems [34,35].

For stay-cable vibration monitoring, FBG sensors can be used to capture dynamic strain responses induced by cable vibration [36,37]. Although an FBG sensor does not directly measure acceleration, the dynamic strain response of a vibrating cable contains modal information that can be used to identify dominant frequencies and spectral characteristics. Therefore, FBG sensing has potential for dynamic characterization of stay cables, especially when frequency-domain features rather than absolute time-domain amplitudes are of primary interest. Several studies have demonstrated the feasibility of FBG-based sensors for dynamic monitoring of civil structures, including bridges and cable-supported systems [38,39]. These studies indicate that FBG sensors can provide useful information for identifying natural frequencies and other vibration-related characteristics [40].

Despite these advantages, FBG-based dynamic monitoring also involves several practical challenges [41,42]. Since FBG sensors are sensitive to both strain and temperature, temperature compensation may be required, particularly for long-term field applications. The measured response may also be affected by bonding quality, packaging configuration, gauge length, sensor location, and interrogator sampling frequency. In addition, when FBG sensors are used as strain sensors, they do not measure the same physical quantity as accelerometers. A PCB accelerometer measures acceleration, whereas an FBG sensor typically measures dynamic strain or wavelength shift associated with strain [43]. Consequently, direct comparison of raw signal amplitudes may be inappropriate unless a reliable transformation or calibration relationship is established.

Because of this difference, a meaningful comparison between FBG sensors and PCB accelerometers should focus on dynamic characteristics that can be extracted consistently from both sensing systems. Parameters such as dominant natural frequencies, spectral peak locations, frequency stability, coherence, and signal consistency provide a more appropriate basis for comparative assessment than direct time-domain amplitude comparison. Both acceleration and dynamic strain responses of the same vibrating cable should contain consistent modal frequency information, even though their amplitudes, phases, and sensitivities may differ.

Although previous studies have separately demonstrated the effectiveness of accelerometers and FBG sensors in structural vibration monitoring, direct field-based comparative assessments between FBG wavelength-shift/dynamic-strain measurements and PCB acceleration measurements on full-scale stay cables under operational bridge conditions remain limited. This gap is important because the two sensing systems measure different physical quantities and therefore cannot be appropriately evaluated through direct time-domain amplitude comparison. A physically meaningful comparison should instead focus on dynamic characteristics that can be consistently extracted from both acceleration-based and strain-based responses, such as dominant modal frequencies, spectral peak locations, frequency deviation, and modal stability.

This study addresses this gap by conducting field measurements on selected stay cables of My Thuan Bridge using both PCB accelerometers and FBG sensors under normal traffic and ambient excitation. The comparison was performed using frequency-domain and modal indicators, including dominant frequency agreement, absolute and relative frequency differences, spectral peak consistency, SSI-COV modal stability, and practical field applicability.

The novelty of this work lies in four main aspects. First, it provides a direct experimental comparison between PCB acceleration responses and FBG wavelength-shift/dynamic-strain responses obtained from full-scale stay cables under in-service bridge conditions. Second, it establishes a frequency-domain comparison strategy that explicitly accounts for the different physical quantities measured by the two sensing systems, avoiding inappropriate direct amplitude equivalence. Third, it combines spectral analysis and SSI-COV stabilization diagrams to evaluate the consistency and reliability of the identified cable vibration frequencies. Fourth, it discusses the practical implications of using FBG sensors as an alternative or complementary sensing technique for stay-cable vibration monitoring, while recognizing that frequency components detected only by the FBG system require further validation. The main contributions of this study are therefore summarized as follows:

(1) Field vibration data from PCB accelerometers and FBG sensors installed on selected stay cables of a full-scale cable-stayed bridge are presented;
(2) A physically consistent comparison between acceleration-based and strain/wavelength-shift-based measurements is performed in the frequency domain;
(3) The agreement between the two sensing systems is quantified using common modal frequencies, absolute and relative frequency differences, and spectral peak consistency;
(4) SSI-COV stabilization diagrams are used to support the interpretation of the identified frequency components; and
(5) The field applicability, advantages, and limitations of FBG sensing for stay-cable dynamic characterization are discussed in comparison with conventional PCB accelerometers.

## 2. Comparative Assessment Framework

This section introduces the conceptual basis used to compare the performance of PCB accelerometers and FBG sensors for field-based dynamic characterization of stay cables. The framework is presented before the experimental setup in order to clarify the comparison logic adopted in this study. Since the two sensing systems measure different physical quantities, acceleration for the PCB accelerometer and wavelength shift associated with dynamic strain for the FBG sensor, direct comparison of raw time-domain amplitudes is not physically appropriate. Therefore, the comparison framework first defines the frequency-domain and modal indicators used in the assessment. The detailed sensor specifications, installation conditions, data-acquisition parameters, and field measurement procedure are subsequently described in Section 3.

**Figure 1:**
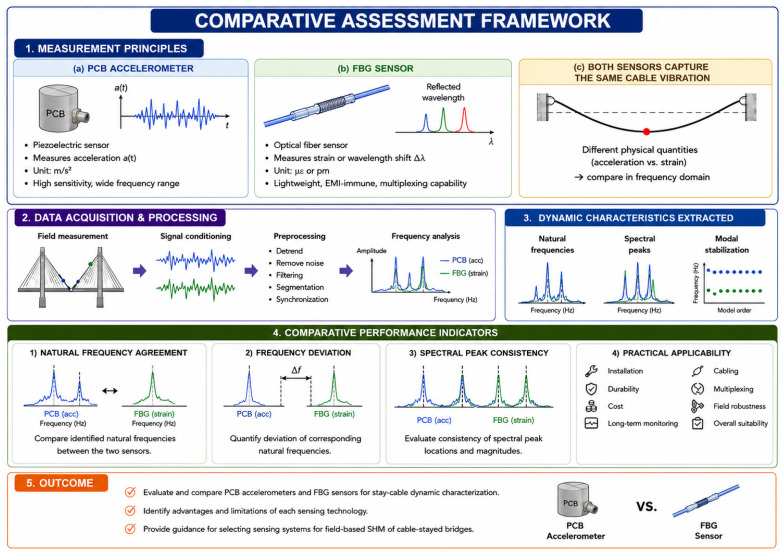
Comparative assessment framework for evaluating PCB accelerometers and FBG sensors in field-based stay-cable dynamic characterization.

Figure 1 presents the comparative assessment framework used to evaluate PCB accelerometers and FBG sensors for field-based dynamic characterization of stay cables. The framework begins with the different measurement principles of the two sensing systems. The PCB accelerometer measures acceleration responses, whereas the FBG sensor measures dynamic strain or wavelength shift. Although the time-domain amplitudes of the two signals are not directly comparable, both responses are generated by the same cable vibration and can therefore provide comparable frequency-domain information.

The measured responses are processed through signal conditioning, preprocessing, synchronization, and frequency-domain analysis. The extracted dynamic characteristics mainly include dominant natural frequencies and spectral peak locations. These quantities are then used to compare the performance of the two sensing systems. The assessment focuses on natural frequency agreement, frequency deviation, spectral peak consistency, and practical field applicability. This framework provides a physically appropriate basis for comparing PCB and FBG measurements without assuming direct equivalence between acceleration and strain responses.

### 2.1. Measurement Principles of PCB Accelerometers and FBG Sensors

PCB accelerometers are piezoelectric sensors that measure acceleration responses induced by structural vibration [44]. When a stay cable vibrates, the inertial force generated inside the accelerometer produces an electrical signal proportional to the acceleration of the mounting point. Because of their high sensitivity, broad frequency range, and mature application in experimental modal analysis, PCB accelerometers are commonly used as reference sensors in vibration testing and dynamic identification. The acceleration response obtained from a PCB accelerometer can be directly analyzed in the time and frequency domains to identify dominant vibration components and modal frequencies.

In contrast, FBG sensors are optical fiber sensors that measure changes in reflected wavelength caused by strain and temperature variations [31]. When an FBG sensor is bonded or attached to a vibrating cable, the dynamic deformation of the cable induces strain in the optical fiber, resulting in a wavelength shift. Therefore, the primary output of an FBG sensor is not acceleration but wavelength variation, which can be converted into dynamic strain if proper calibration is available. Although this measured quantity differs from acceleration, the dynamic strain response still contains frequency information associated with cable vibration.

The fundamental difference between the two sensing systems is therefore the measured physical response. The PCB accelerometer records acceleration at the sensor location, whereas the FBG sensor captures local dynamic strain or wavelength shift [45]. As a result, the amplitudes, phases, and sensitivities of the two signals may differ even when both sensors are installed on the same cable. However, if both sensors capture the vibration response of the same stay cable, their frequency-domain responses should contain consistent dominant modal components.

### 2.2. Basis for Comparing Acceleration-Based and Strain-Based Responses

For a vibrating stay cable, acceleration and strain responses are different manifestations of the same dynamic motion. Acceleration is associated with the second derivative of displacement with respect to time, whereas strain is related to local deformation of the cable. Therefore, the two responses are not expected to be identical in the time domain. Their amplitudes may differ significantly because of sensor type, installation location, measurement direction, gauge length, bonding condition, and sensitivity to local deformation.

Because of these differences, this study does not aim to establish direct amplitude equivalence between the PCB and FBG measurements. Instead, the comparison focuses on dynamic features that are physically meaningful and can be extracted from both signals. In particular, the dominant natural frequencies of the stay cable are expected to appear in both acceleration and dynamic strain spectra. Therefore, frequency-domain analysis provides an appropriate basis for evaluating whether the FBG sensor can capture the main dynamic characteristics of the stay cable in a manner consistent with the PCB accelerometer.

The PCB accelerometer is considered as a conventional reference sensor because accelerometers are widely used in field vibration testing and modal identification. The FBG sensor is evaluated against the PCB accelerometer in terms of its ability to identify dominant frequency components, reproduce spectral peak locations, and provide consistent frequency-domain information under field measurement conditions.

### 2.3. Performance Indicators for Dynamic Characterization

To compare the performance of the PCB accelerometer and FBG sensor, a set of frequency-based and practical indicators was used. These indicators were selected to evaluate the capability of each sensing system to capture the dominant dynamic characteristics of stay cables under field conditions. Since the PCB accelerometer measures acceleration, while the FBG sensor measures dynamic strain or wavelength shift, the comparison was focused on frequency-domain characteristics rather than direct time-domain amplitude equivalence.

The first indicator is natural frequency agreement. The dominant frequencies identified from the PCB and FBG measurements were compared mode by mode. A frequency component was considered common when it was detected by both sensing systems at approximately the same spectral location. The signed frequency difference for the i-th common peak is defined as:

(1) $$e_{f,i}=f_{i,\mathrm{FBG}}-f_{i,\mathrm{PCB}}$$

where $f_{i,\mathrm{FBG}}$ and $f_{i,\mathrm{PCB}}$ are the modal frequencies identified from the FBG sensor and PCB accelerometer, respectively. The absolute frequency difference is calculated as:

(2) $$\Delta f_{i}=\left|f_{i,\mathrm{FBG}}-f_{i,\mathrm{PCB}}\right|$$

Since this study aims to compare two sensing systems rather than to assume one measurement as the exact reference, the relative frequency difference is calculated using the average value of the two identified frequencies:

(3) $$\delta f_{i}=\frac{\left|f_{i,\mathrm{FBG}}-f_{i,\mathrm{PCB}}\right|}{\left(f_{i,\mathrm{FBG}}+f_{i,\mathrm{PCB}}\right)/2}\times 100\%$$

This symmetric definition avoids assigning absolute reference status to either sensing system and provides a balanced measure of agreement between the PCB and FBG measurements.

The second indicator is spectral peak consistency. This indicator evaluates whether the dominant peaks observed from one sensing system also appear in the other. Since the two sensors measure different physical quantities, their spectral amplitudes are not expected to be identical. Therefore, the comparison focuses mainly on the locations of spectral peaks rather than their magnitudes. Consistent peak locations indicate that both sensing systems capture the same vibration characteristics of the stay cable.

To quantify spectral peak consistency, a directional matching ratio was first defined to evaluate the proportion of FBG-detected frequency components that were also identified at corresponding spectral locations in the PCB accelerometer measurements. This index reflects the extent to which the frequency components obtained from the FBG response are consistent with those from the conventional accelerometer measurement. It is referred to in this study as the FBG-confirmed-by-PCB ratio and is expressed as:

(4) $$R_{\mathrm{FBG}|\mathrm{PCB}}=\frac{N_{\mathrm{common}}}{N_{\mathrm{FBG}}}\times 100\%$$

where $N_{\mathrm{common}}$ is the number of frequency components identified by both sensing systems and $N_{\mathrm{FBG}}$ is the total number of dominant frequency components identified from the FBG measurement.

To avoid privileging either sensing system, a symmetric similarity index was also introduced using the Dice similarity coefficient:

(5) $$R_{D}=\frac{2N_{\mathrm{common}}}{N_{\mathrm{PCB}}+N_{\mathrm{FBG}}}\times 100\%$$

where $N_{\mathrm{PCB}}$ and $N_{\mathrm{FBG}}$ are the numbers of dominant frequency components identified from the PCB and FBG measurements, respectively. This coefficient accounts for the number of peaks detected by both systems and provides a balanced measure of PCB–FBG spectral similarity.

For each measurement location, the mean absolute frequency difference was calculated as:

(6) $$\bar{\Delta f}=\frac{1}{N_{\mathrm{common}}}\sum_{i=1}^{N_{\mathrm{common}}}\Delta f_{i}$$

Similarly, the mean relative frequency difference was calculated as:

(7) $$\bar{\delta f}=\frac{1}{N_{\mathrm{common}}}\sum_{i=1}^{N_{\mathrm{common}}}\delta f_{i}$$

These two indicators provide location-level measures of the average agreement between the PCB and FBG measurements. In addition, the maximum absolute and relative differences were used to identify the largest discrepancy between the two sensing systems at each location:

(8) $$\Delta f_{\max}=\max\left(\Delta f_{i}\right)$$

(9) $$\delta f_{\max}=\max\left(\delta f_{i}\right)$$

The third indicator is practical field applicability. In addition to frequency-based comparison, the practical suitability of each sensing system was also considered. This includes installation requirements, cabling, robustness under field conditions, multiplexing capability, and suitability for long-term monitoring. This practical assessment is important because field-based stay-cable monitoring depends not only on frequency identification accuracy but also on the feasibility of deploying and maintaining the sensing system under operational bridge conditions. In summary, the comparative assessment in this study focuses on three main aspects: the agreement of identified dominant frequencies, the consistency of spectral peak detection, and the practical applicability of PCB accelerometers and FBG sensors for stay-cable dynamic monitoring.

## 3. Field Measurement and Dynamic Identification

This section describes the field measurement program and the signal processing procedure used for identifying the dynamic characteristics of the stay cable. The measurement program includes the description of the tested bridge and cable, sensor installation, data acquisition system, and field-testing conditions. The measured responses from the PCB accelerometer and FBG sensor are then preprocessed and analyzed in the frequency domain to extract the dominant dynamic characteristics of the stay cable. Since the two sensing systems measure different physical quantities, the identification procedure focuses primarily on frequency-domain features, including dominant spectral peaks and natural frequencies, rather than direct comparison of time-domain amplitudes.

### 3.1. Description of the Tested Bridge and Stay Cable

**Figure 2:**
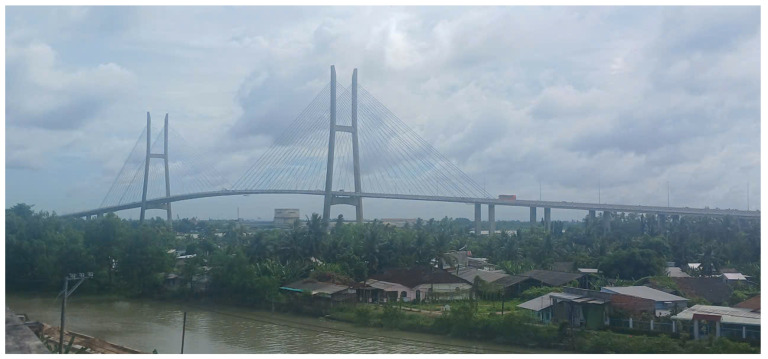
My Thuan Bridge.

The field measurement was conducted on stay cables of My Thuan Bridge (Figure 2). My Thuan Bridge located on National Highway 1A and connecting Tien Giang and Vinh Long provinces, Vietnam. The bridge has a total length of 1535.2 m, including a 650 m cable-stayed main bridge and an 875.2 m approach bridge. The main bridge consists of three continuous spans with a span arrangement of 150 m + 350 m + 150 m. The bridge deck accommodates four traffic lanes and two pedestrian lanes, with a total width of 23.66 m. These structural characteristics make My Thuan Bridge a representative cable-stayed bridge for field-based vibration monitoring and dynamic characterization.

The stay-cable system of My Thuan Bridge is arranged in a semi-harp configuration with two cable planes spaced 18.6 m apart. The cable pairs on the upstream and downstream sides are symmetrically arranged with respect to the vertical plane passing through the bridge centerline. The inclination angle of the stay cables varies from 31.031° for the outermost cable to 77.39° for the cable closest to the pylon in the side span. In total, the bridge has 128 stay cables divided into eight groups, with 16 cables in each group. The stay cables consist of Freyssinet seven-wire strands with a nominal strand diameter of 15.2 mm, and the number of strands varies depending on cable location. The outermost side-span cable contains the largest number of strands, while the main-span cable closest to the pylon contains the smallest number of strands.

**Figure 3:**
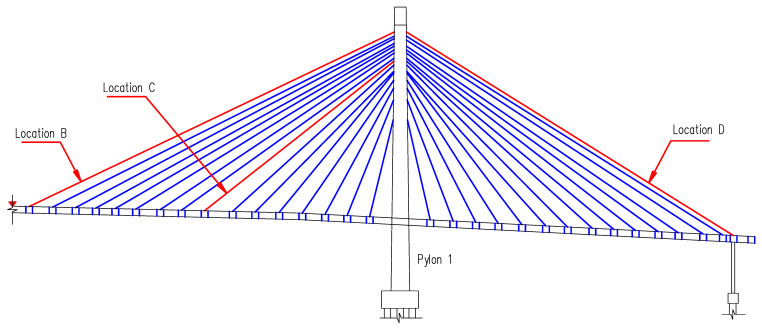
Layout of field measurement locations on My Thuan Bridge.

The selected measurement locations were chosen to represent different vibration response characteristics within the stay-cable system (Figure 3). Locations B, C, and D were arranged on selected stay cables to capture local cable vibration responses under field operating conditions. Specifically, Locations B and D were positioned on the two outermost stay cables of the cable plane, whereas Location C was positioned on the 8th stay cable counted from pylon 1. These selected cables correspond to different positions within the stay-cable system and may therefore exhibit different dynamic characteristics due to variations in cable length, inclination, boundary conditions, and interactions with the bridge deck and pylon. The two outermost cables are expected to represent the dynamic behavior of long and flexible stay cables, while the 8th cable provides an intermediate case within the same cable system. This measurement arrangement provides a suitable basis for evaluating the ability of PCB accelerometers and FBG sensors to capture the dynamic responses of stay cables under different field conditions. By considering selected cables with different structural characteristics, the field test enables a comparative assessment of the two sensing systems in identifying local cable vibration features, including dominant frequencies, frequency deviation, spectral peak consistency, and modal stabilization behavior.

### 3.2. Field Testing

The field testing program was designed to obtain vibration responses from selected stay cables under actual operating conditions. During the measurements, the bridge remained open to normal traffic, and no artificial excitation was applied. The recorded responses were therefore mainly induced by ambient sources, traffic loading, wind action, and the interaction between the bridge deck and the stay-cable system. This test condition reflects the bridge’s in-service vibration behavior and provides a practical basis for evaluating sensing performance under field conditions.

At each measurement location, the PCB accelerometer and FBG sensing system were installed to record dynamic responses during the same testing period. The PCB accelerometer measured acceleration responses, while the FBG sensor recorded dynamic strain or wavelength-shift responses. The two measurement systems were arranged to capture comparable vibration responses at the selected structural components. Particular attention was paid to sensor attachment, cable protection, and measurement stability, as these factors may affect signal quality, especially in field environments. The data-acquisition procedure was conducted for a sufficient duration to capture representative vibration responses under operational excitation. For each test case, the raw signals from the PCB accelerometer and FBG sensor were recorded simultaneously or under closely comparable testing conditions. The main acquisition parameters, including sampling frequency, measurement duration, sensor direction, and data format, were documented for subsequent signal processing and comparison. When necessary, the recorded signals were synchronized before being analyzed in the time and frequency domains.

The field testing program was intended not to compare the raw amplitudes of the PCB and FBG signals directly, but to evaluate whether both sensing systems could identify consistent dynamic characteristics from the same bridge and stay-cable responses. The collected data were used for signal preprocessing, spectral analysis, modal peak identification, and comparative assessment based on frequency-domain indicators. The PCB and FBG systems were not hardware-synchronized through a common trigger. Instead, the measurements were conducted at closely comparable time periods under similar operational conditions. Since the objective of this study was to compare frequency-domain characteristics rather than time-domain amplitude equivalence, the comparison was based on dominant frequency components, spectral peak locations, and SSI-COV stabilization behavior extracted from records with the same acquisition duration and sampling rates sufficient for the investigated frequency range.

The acceleration response of the stay cables was measured using PCB 393B12 accelerometers connected to a National Instruments CompactDAQ acquisition system (supplied by UCT Company, Hanoi, Vietnam). The PCB 393B12 is a high-sensitivity seismic ICP^®^ accelerometer suitable for low-amplitude structural vibration measurements. According to the manufacturer’s specifications, the accelerometer has a nominal sensitivity of 10,000 mV/g, a measurement range of 0.5 g pk, and a frequency range of 0.15–1000 Hz within ±5%. Detailed specifications are provided in Table 1.

Commercial FBG sensors supplied by FBG Korea (Daejeon, Republic of Korea), model FBG-ST-310, were used in this study. Each FBG sensor consisted of a Bragg grating inscribed in a single-mode optical fiber and was protected with a field-ready packaging to ensure stable attachment to the cable surface during vibration measurements. The main optical and sensing parameters of the FBG sensors are summarized in Table 2.

Because the PCB accelerometer acts as a point sensor whereas the FBG sensor measures strain over a finite bonded length, their relative installation positions on each tested stay cable are clarified in Table 3.

The PCB accelerometer and FBG sensor were installed within the same local measurement zone but were not exactly co-located. This arrangement was necessary because the PCB accelerometer acts as a point sensor, whereas the FBG sensor measures the strain response over a finite bonded length.

**Figure 4:**
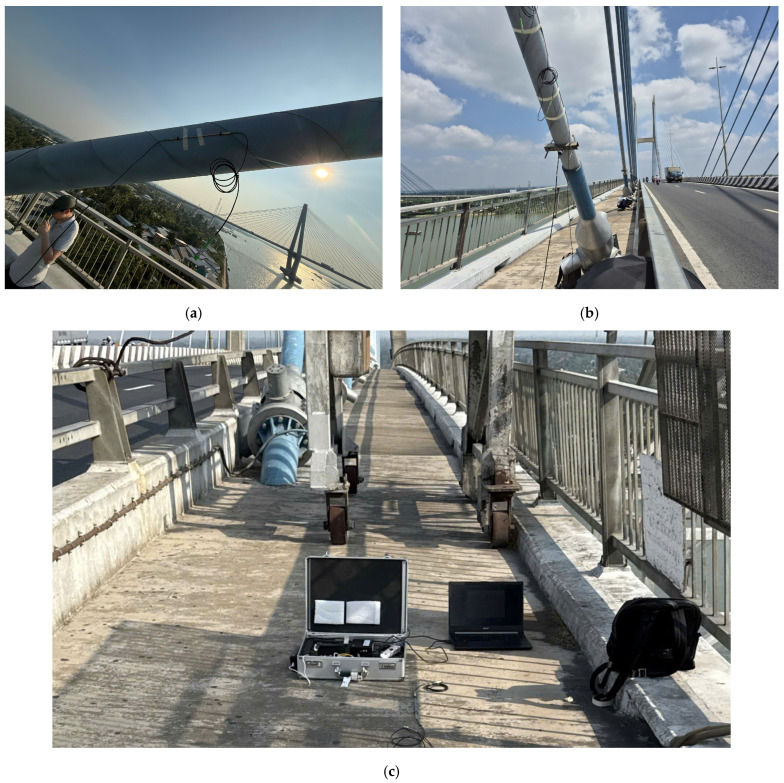
Field measurements: (**a**) using FBG; (**b**) using PCB; (**c**) Data-acquisition station.

For each tested cable, the PCB accelerometer and FBG sensor were installed at the same cable segment (Figure 4). The measurement position was selected sufficiently away from the anchorage and damping devices to reduce local boundary effects on the measured vibration response. This arrangement was adopted to ensure that the recorded signals reflected the representative vibration behavior of the cable rather than local effects near the cable ends. The PCB accelerometer was used to record acceleration responses, while the FBG sensor was used to measure dynamic strain or wavelength-shift responses. The recorded data were synchronized before subsequent signal processing and comparison.

### 3.3. Signal Processing and Dynamic Identification

The vibration responses measured by the PCB accelerometers and FBG sensors were processed using a unified signal processing procedure to identify the dominant dynamic characteristics of the selected stay cables. Since the two sensing systems measure different physical quantities, acceleration for the PCB accelerometer and dynamic strain or wavelength shift for the FBG sensor, the analysis focused on frequency-domain and modal characteristics rather than direct comparison of time-domain amplitudes.

The raw signals were first inspected to identify abnormal records, signal interruptions, or obvious measurement noise. The mean value of each signal was removed, and detrending was applied to reduce low-frequency drift. When necessary, filtering was used to retain the frequency range associated with stay-cable vibration and to suppress irrelevant low-frequency and high-frequency components. In the present study, no independent temperature-compensation FBG or temperature sensor was installed during the short-term dynamic tests. Therefore, the FBG response was not used to evaluate absolute static strain or long-term strain variation. Instead, the analysis focused only on the dynamic frequency content of the measured wavelength-shift response. Slow thermal drift and low-frequency baseline variations were reduced through mean removal, linear detrending, and band-pass filtering before spectral analysis and SSI-COV identification.

The signal normalization used in this study was applied only for plotting and numerical conditioning during frequency-domain processing. It was not used to establish time-domain equivalence between acceleration and strain responses, and no normalized waveform amplitude comparisons were used for performance evaluation. Since PCB accelerometers measure acceleration, whereas FBG sensors measure wavelength shift associated with dynamic strain, the two signals are not directly comparable in the time domain. Accordingly, the comparison was restricted to frequency-domain indicators, including dominant frequency agreement, spectral peak locations, frequency deviation, and SSI-COV stabilization behavior.

For both PCB and FBG records, the same signal-processing parameters were used to ensure consistency in the comparative assessment. After mean removal and linear detrending, the signals were filtered using a fourth-order Butterworth band-pass filter with cut-off frequencies of 0.05 Hz and 30 Hz. This range was selected to retain the relevant stay-cable vibration components observed in the measured data while suppressing very-low-frequency drift and high-frequency noise. Zero-phase forward–backward filtering was applied to avoid phase distortion. The frequency-domain characteristics were then examined using the Fast Fourier Transform (FFT) and power spectral density (PSD) estimation. The FFT was computed using the full 10-min record. The PCB and FBG systems used different sampling frequencies, 2048 Hz and 13,336.9 Hz, respectively, but both were sufficient for the investigated frequency range below 30 Hz. The PSD was estimated using Welch’s method with a Hann window, a 60-s segment length, 50% overlap, and an FFT length equal to the segment length, giving a PSD frequency resolution of approximately 0.0167 Hz. These processing parameters were applied identically to the PCB acceleration and FBG wavelength-shift responses before modal identification and frequency comparison.

**Figure 5:**
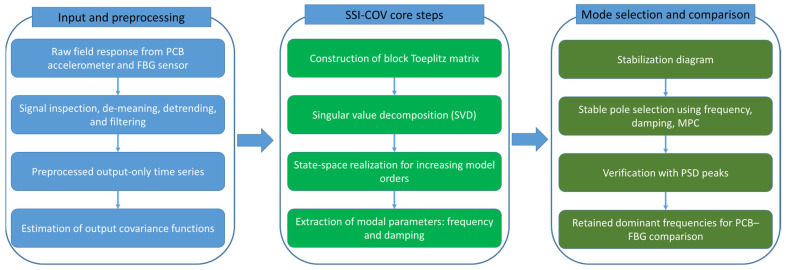
Workflow of the SSI-COV-based modal identification procedure used for PCB and FBG measurements.

To support modal identification and reduce the possibility of selecting noise-induced spectral peaks, the covariance-driven stochastic subspace identification method (SSI-COV) was applied to the preprocessed output responses. The SSI-COV procedure adopted in this study is illustrated in Figure 5. First, the preprocessed acceleration or wavelength-shift response was arranged as an output-only time-series signal. The output covariance functions were then estimated for a prescribed number of time lags. These covariance matrices were used to construct a block Toeplitz matrix, which contains the correlation information of the measured response. Singular value decomposition was subsequently applied to the block Toeplitz matrix to obtain a reduced-order state-space realization of the dynamic system. From the estimated state matrix, the modal parameters, including natural frequencies and damping ratios, were extracted for increasing model orders.

In this study, the SSI-COV analysis was performed using model orders from 10 to 80. Stabilization diagrams were generated by plotting the identified poles against increasing model orders. A pole was classified as stable when it appeared persistently over consecutive model orders, with a relative frequency variation lower than 1%, a damping-ratio variation lower than 10%, and a positive damping ratio within the physically meaningful range of 0–10%. Since the present analysis was mainly based on single-response measurements at each sensor location, the MAC criterion was not used as a primary stabilization criterion. The final retained frequencies were selected when stable poles were also supported by recognizable PSD peaks.

For the present single-response or limited-response measurements, the primary stabilization criteria were frequency persistence and physically reasonable damping. The final dominant frequencies were selected using a combined spectral and SSI-COV criterion. A frequency component was retained when it appeared as a stable pole in the SSI-COV stabilization diagram and was also supported by a recognizable peak in the PSD spectrum. This combined criterion was adopted to avoid relying on peak picking alone and to reduce the influence of random noise, weak harmonic components, and numerical artifacts. The retained poles were then used to construct the final frequency lists for the PCB and FBG measurements. These identified frequencies were compared mode by mode using absolute frequency difference, relative frequency difference, and spectral peak consistency.

## 4. Results and Discussion

### 4.1. Identification and Comparison of Dominant Dynamic Frequencies

The dominant dynamic frequencies of the selected stay cables were identified from both PCB accelerometer and FBG sensor measurements. For each measurement location, the preprocessed responses were analyzed using frequency-domain analysis and SSI-COV modal identification. The dominant frequency components were selected based on the consistency between spectral peaks and stable poles obtained from the SSI-COV procedure. This combined criterion was adopted to reduce the possibility of selecting noise-induced peaks and to improve the reliability of the identified dynamic characteristics.

Table 4 summarizes the dominant dynamic frequencies identified from the PCB accelerometer and FBG sensor measurements at the selected stay-cable locations. The comparison was conducted for Locations B, C, and D, where Locations B and D correspond to the two outermost stay cables of the cable plane, and Location C corresponds to the 8th stay cable counted from pylon 1. The identified frequencies were obtained from the combined use of frequency-domain analysis and SSI-COV modal identification. Only frequency components that were supported by clear spectral peaks or stable SSI-COV poles were retained as dominant dynamic frequencies.

For Location B, the FBG sensor identified 13 dominant frequency components in the range from 2.132 Hz to 23.450 Hz, whereas the PCB accelerometer identified six corresponding components. Among the common frequencies detected by both sensing systems, very good agreement was observed. The relative differences ranged from 0.00% to 0.31%, with the largest difference occurring at the third identified peak. The identical frequency at 2.826 Hz and the small deviations at 3.510–3.521 Hz, 4.188–4.178 Hz, 9.963–9.953 Hz, 12.111–12.131 Hz, and 18.094–18.124 Hz indicate that the FBG sensor was able to capture the dominant vibration characteristics of the outermost cable consistently with the PCB accelerometer.

For Location C, a larger number of common frequency components was identified from both sensors. The FBG sensor detected 19 dominant frequencies, while the PCB accelerometer provided corresponding values for 17 components. The common frequencies covered a wide range from approximately 2.231 Hz to 19.091 Hz. Most relative differences were lower than 2%, indicating good consistency between the two sensing systems. The lowest differences were observed at 2.231 Hz, 7.449–7.451 Hz, 12.802 Hz, and 13.808–13.806 Hz. The maximum relative difference at this location was 3.51%, corresponding to the seventh identified peak. This discrepancy may be related to differences in sensor type, local strain sensitivity, mounting condition, or peak selection under closely spaced spectral components.

For Location D, the FBG sensor identified 21 dominant frequency components, while the PCB accelerometer identified six corresponding components. Good agreement was obtained for the first four common peaks, with relative differences ranging from 0.15% to 1.09%. Larger differences were observed at the 12th and 13th identified peaks, with relative differences of 4.26% and 3.44%, respectively. These higher deviations may indicate reduced spectral clarity, local measurement effects, or differences in the sensitivity of the two sensors at higher-frequency components.

The results in Table 5 show that the FBG sensor can capture the dominant dynamic frequencies of the selected stay cables with good agreement relative to the PCB accelerometer. Considering all frequency components identified by both sensing systems, the relative difference ranges from 0.00% to 4.26%, with an average value of approximately 0.94%. This confirms that the frequency-domain information obtained from the FBG sensor is generally consistent with that obtained from the PCB accelerometer. Although several frequency components were detected only in the FBG measurements, these results should be interpreted with caution and further examined through spectral consistency and stabilization analysis. Nevertheless, the strong agreement for the common identified frequencies demonstrates the feasibility of using FBG sensing for field-based dynamic characterization of stay cables.

Table 5 summarizes the agreement between the PCB and FBG measurements using both directional and symmetric matching metrics. Location C shows the strongest consistency between the two sensing systems. The highest FBG-confirmed-by-PCB ratio of 89.47%, a Dice similarity coefficient of 94.44%, and a mean relative frequency difference of 0.99%. Location B has a lower FBG-confirmed-by-PCB ratio of 46.15%, but the matched peaks show very small deviations, with a mean relative difference of 0.16% and a maximum of 0.31%. Location D presents the lowest FBG-confirmed-by-PCB ratio of 28.57% and the largest maximum relative difference of 4.26%, indicating greater discrepancy between the two sensing systems at this location. 29 common peaks were identified from 29 PCB-detected peaks and 53 FBG-detected peaks, resulting in an FBG-confirmed-by-PCB ratio of 54.72% and a Dice similarity coefficient of 70.73%. The overall mean absolute difference was 0.093 Hz, and the overall mean relative difference was 0.94%. These results indicate that the PCB and FBG measurements provide good frequency agreement for the common detected components.

At Locations B and D, the PCB accelerometer identified only six common components at each location, whereas the FBG sensor detected 13 and 21 components, respectively. These discrepancies should not be interpreted as direct evidence of superior FBG performance. Most of the FBG-only components fall within the investigated stay-cable vibration frequency range. Therefore, they are unlikely to be missing from the PCB results simply because they are outside the accelerometer bandwidth. In addition, the PCB accelerometer acts as a point sensor, measuring acceleration in a specific direction, whereas the FBG sensor measures local dynamic strain over a finite bonded length. Therefore, some vibration components may be more clearly expressed in the strain response than in the local acceleration response. Some weak PCB components may also have been rejected during the SSI-COV procedure because they did not form sufficiently persistent, stable poles or lacked clear PSD peaks. For these reasons, the additional FBG-only components are interpreted as candidate modal-related frequency components rather than independently validated vibration modes.

The identified frequencies were also examined in relation to the expected vibration behavior of stay cables. For an ideal taut cable, the modal frequencies are approximately distributed according to a harmonic pattern, with higher-order frequencies nearly proportional to the mode order. Although actual stay cables may deviate from this ideal behavior due to sag, bending stiffness, boundary conditions, damping devices, and deck–cable interaction, a nearly regular frequency spacing is still expected for the dominant cable vibration components.

The FBG results show a frequency distribution that is generally consistent with this cable-vibration characteristic. At Location B, the identified FBG frequencies form a nearly regular sequence from 2.132 Hz to 6.896 Hz, with a spacing of approximately 0.68–0.70 Hz. Higher-frequency components at 9.953 Hz, 12.131 Hz, 18.124 Hz, 22.911 Hz, and 23.450 Hz also appear compatible with higher-order cable modes, although some intermediate modes may not be clearly captured by the PCB accelerometer. At Location C, the identified frequencies increase almost regularly from 2.231 Hz to 19.091 Hz, with a spacing of approximately 1 Hz, which is reasonable for a shorter or stiffer cable compared with the outermost cables. At Location D, the frequencies from 2.911 Hz to 14.945 Hz also show a nearly uniform progression, indicating a consistent modal pattern.

This observation suggests that the dominant frequencies identified from the FBG measurements are not random spectral peaks but are physically consistent with the expected dynamic behavior of stay cables. The PCB accelerometer confirms several of these dominant components, particularly at the common peaks. However, the FBG sensor detects additional frequency components that also follow the cable-frequency progression. This indicates that FBG sensing may provide complementary frequency-domain information for stay-cable dynamic characterization, although the FBG-only components require further validation.

### 4.2. SSI-COV Stabilization Diagrams

The reliability of the identified frequency components was further examined using the SSI-COV stabilization diagrams. This assessment was performed to verify whether the frequencies summarized in Table 5 correspond to stable modal components of the stay cables rather than numerical poles or random noise. In the stabilization diagrams, physically meaningful modes are expected to appear as stable vertical alignments across increasing model orders. Therefore, the persistence of stable poles was used as the main criterion for confirming the reliability of the identified frequencies.

**Figure 6:**
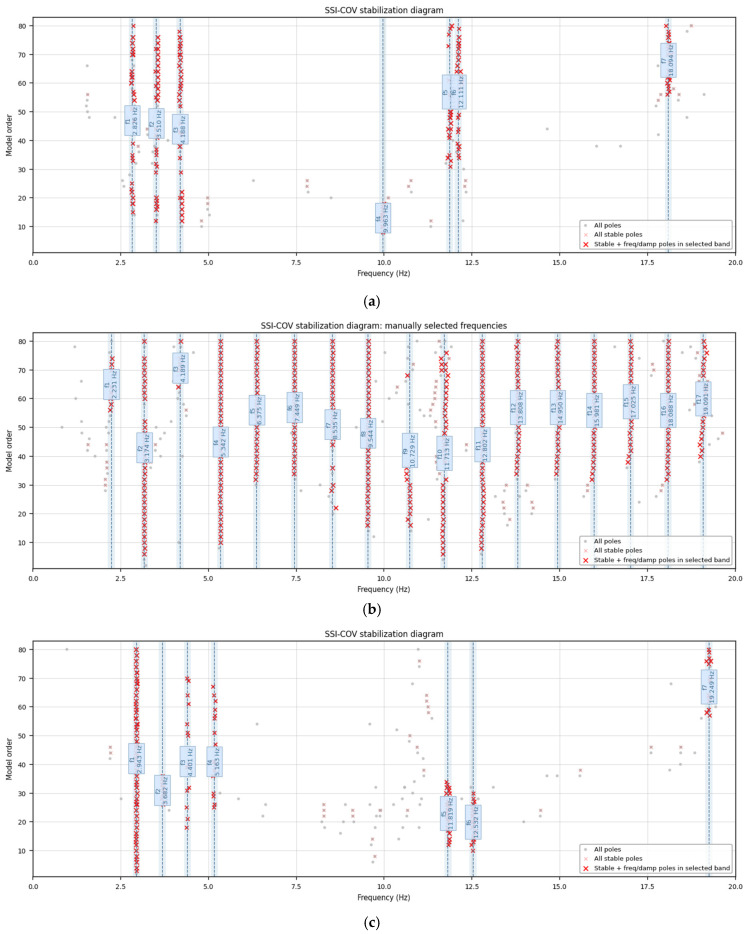
SSI-COV stabilization diagrams for PCB measurements: (**a**) Location B; (**b**) Location C; (**c**) Location D.

For the PCB measurements, the SSI-COV stabilization diagrams show that the accelerometer mainly captures the strongest vibration components of the selected stay cables (Figure 6). At Location B, stable poles are concentrated around a limited number of frequencies, indicating that only the most dominant cable modes are clearly identified. A similar pattern is observed at Location D, where the stable components are also limited in number. In contrast, Location C exhibits a richer sequence of stable poles, suggesting that the cable response at this location is more clearly represented in the acceleration measurements. The PCB results provide reliable identification of the dominant cable vibration frequencies, although some weaker or higher-order components are not clearly captured.

**Figure 7:**
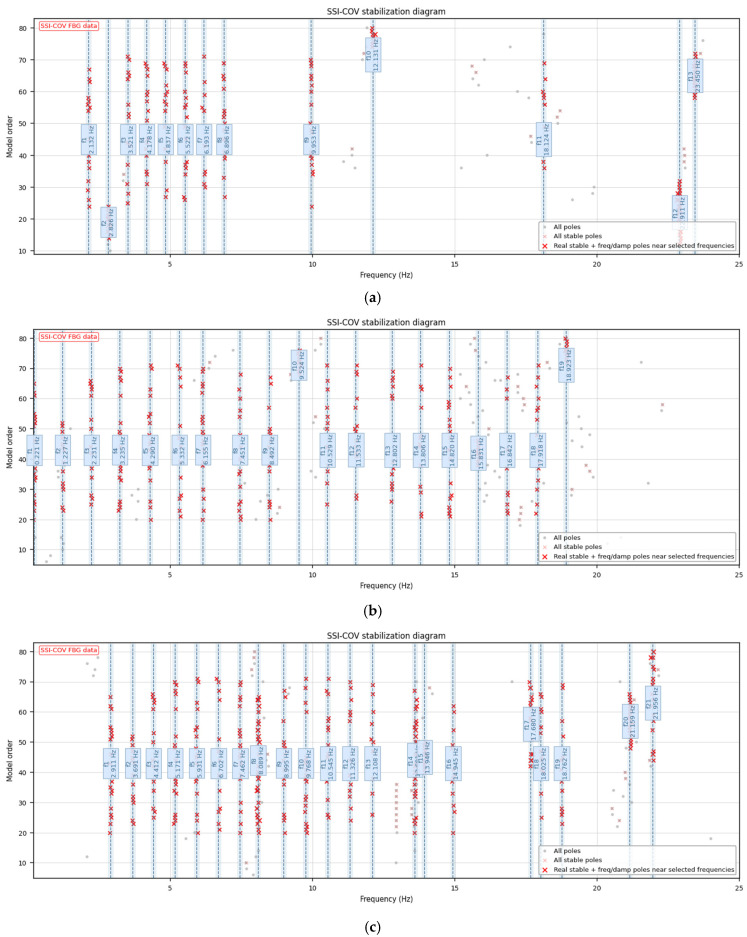
SSI-COV stabilization diagrams for FBG measurements: (**a**) Location B; (**b**) Location C; (**c**) Location D.

For the FBG measurements, the SSI-COV stabilization diagrams reveal a denser distribution of stable frequency components over the investigated frequency range (Figure 7). At Locations B, C, and D, several stable poles appear across multiple model orders, indicating that the FBG sensor can capture not only the dominant cable modes but also additional weaker frequency components. The FBG result at Location C shows a particularly clear and regular progression of stable components, while Locations B and D also present multiple stable poles consistent with stay-cable vibration behavior. These observations suggest that FBG sensing provides complementary frequency-domain information for stay-cable dynamic characterization.

Among the three measurement locations, Location C exhibits the clearest consistency between the PCB and FBG results. Both sensing systems show a relatively continuous sequence of stable components, indicating that the identified frequencies at this location are strongly supported by the SSI-COV modal identification. Locations B and D show fewer common components, but the stable poles identified by both systems remain consistent for the dominant frequencies detected in both measurements.

The SSI-COV stabilization results confirm the modal reliability of the dominant frequencies identified from both sensing systems. The PCB accelerometer mainly captures the strongest cable vibration components, whereas the FBG sensor provides a richer set of stable frequency components. This indicates that FBG sensing can complement conventional accelerometer-based measurements by revealing additional modal information while maintaining good consistency for the common dominant frequencies.

The additional frequency components detected solely from the FBG measurements are not considered independently validated global vibration modes. Rather, they are treated as candidate modal-related frequency components. Their possible physical relevance was examined using a combined consistency check based on SSI-COV stabilization behavior, PSD support, and the expected frequency progression of stay cables. First, a frequency component was retained only when it appeared as a persistent stable pole in the SSI-COV stabilization diagram over consecutive model orders. Second, the component had to be supported by a recognizable peak in the PSD spectrum, so that isolated numerical poles, random noise-related components, or unstable spectral fluctuations were excluded. Third, the retained frequencies were compared qualitatively with the expected modal progression of stay cables. For an ideal taut cable, the natural frequencies can be approximately expressed as:

(10) $$f_{n}=\frac{n}{2L}\sqrt{\frac{T}{\mu }}$$

where $f_{n}$ is the nth natural frequency, *L* is the cable length, *T* is the cable tension force, and μ is the mass per unit length.

Based on this physical interpretation, the FBG-only frequency components were examined to determine whether they followed an approximately regular frequency progression. For example, the FBG frequencies at Location B showed a nearly regular sequence from 2.132 Hz to 6.896 Hz, while the frequencies identified at Locations C and D also exhibited approximately uniform spacing over the investigated frequency range. This behavior suggests that several of the additional FBG-detected components may be related to stay-cable vibration rather than purely random noise. However, because a fully calibrated analytical cable model or finite element model was not available in the present study, these FBG-only components cannot be conclusively confirmed as physical modes. They may still be influenced by local strain sensitivity, bonding condition, cable–deck interaction, higher-order harmonics, or residual numerical artifacts. Therefore, the main confirmed finding of this study is the good agreement between PCB and FBG measurements for the common identified frequencies. The additional FBG-only components indicate the potential complementary value of strain-based optical sensing for stay-cable dynamic characterization.

### 4.3. Discussion

The results demonstrate that both PCB accelerometers and FBG sensors can provide useful frequency-domain information for stay-cable dynamic characterization. The PCB accelerometer identifies the strongest acceleration-related vibration components and provides reliable reference information for dominant cable frequencies. This behavior is consistent with the conventional use of piezoelectric accelerometers in field vibration testing. However, the number of identified components from the PCB measurements is lower than that from the FBG measurements at several locations, especially for Locations B and D. This indicates that some weaker or higher-order cable vibration components may not be clearly represented in the acceleration response.

Although the FBG sensor measures dynamic strain or wavelength shift rather than acceleration, its identified frequencies show good agreement with the PCB results for the common peaks. More importantly, many additional FBG-detected peaks follow a physically reasonable modal progression of stay cables. This suggests that these components are unlikely to be random noise and may correspond to weaker cable vibration modes that are more visible in the strain-based response. Therefore, FBG sensing can provide complementary dynamic information to conventional accelerometer measurements.

The observed frequency patterns are also consistent with the expected vibration behavior of stay cables. For a stay cable, dominant frequencies generally follow an approximately regular modal progression, although deviations may occur due to sag, bending stiffness, boundary conditions, damping devices, and interaction with the bridge deck and pylon. The FBG results, particularly at Location C, show a relatively continuous sequence of frequency components. This supports the physical reliability of the identified frequencies and confirms that the proposed identification procedure captures meaningful cable vibration characteristics.

From a sensing-performance perspective, the PCB accelerometer remains advantageous for direct acceleration measurement, mature field application, and straightforward interpretation of vibration responses. In contrast, the FBG sensor offers important advantages for long-term SHM applications, including compact size, immunity to electromagnetic interference, corrosion resistance, multiplexing capability, and suitability for distributed or multi-point monitoring. These features are particularly relevant for stay-cable systems, where a large number of cables may need to be monitored over long periods.

However, the use of FBG sensors also requires careful consideration. The quality of FBG-based dynamic measurement may be affected by bonding condition, sensor packaging, gauge length, temperature sensitivity, interrogator sampling rate, and local strain transfer between the cable and the optical fiber. Therefore, FBG sensors should not be evaluated by direct amplitude comparison with accelerometers. A frequency-based comparison, as adopted in this study, provides a more appropriate basis for assessing their performance in dynamic characterization.

The comparison indicates that FBG sensing can be used as an effective complementary technique to PCB accelerometers for field-based stay-cable vibration monitoring. The PCB accelerometer provides reliable identification of dominant acceleration-based frequencies, while the FBG sensor can capture a richer set of strain-related frequency components. The agreement between the common frequencies confirms the consistency of the two sensing systems, whereas the additional FBG-detected components highlight the potential of fiber-optic sensing for enhanced modal information in long-term SHM applications. Table 3 presents the main advantages, limitations, and field applicability of PCB accelerometers and FBG sensors for stay-cable dynamic characterization.

It should be noted that the qualitative ratings in Table 6 are not intended to represent direct quantitative results obtained from the present field test. Instead, they summarize general practical characteristics of PCB accelerometers and FBG sensors reported in the literature and observed during field deployment. Therefore, terms such as “High”, “Moderate”, and “Low” are used only as qualitative descriptors of typical sensor characteristics, including installation requirements, cabling complexity, multiplexing capability, environmental robustness, and suitability for long-term monitoring. The quantitative comparison in this study is provided separately through the frequency agreement indicators, including common identified frequencies, absolute and relative frequency differences, and similarity metrics.

The results obtained in this study were further compared with findings reported in the literature to place the PCB–FBG agreement in a broader context. Previous studies on stay-cable vibration monitoring have commonly used accelerometers as reference sensors because of their accuracy, mature application in modal testing, and ability to directly measure acceleration responses. However, several alternative sensing techniques, including fiber-optic sensors, microwave interferometry, vision-based systems, and optical tiltmeters, have also been investigated for cable or bridge dynamic characterization.

Gentile et al. [46] investigated microwave remote sensing for measuring the dynamic response of stay cables and compared the identified cable frequencies with those obtained from conventional accelerometers. Their results showed that remote sensing techniques can identify a large number of cable frequencies with accuracy comparable to accelerometers, particularly when the objective is to extract local natural frequencies for SHM purposes. Similarly, Xiao et al. [47] used FBG tiltmeters for bridge dynamic-response monitoring and reported that the primary natural frequencies could be extracted with good agreement with reference results, with a maximum difference of approximately 5.3%. Other studies using FBG-based vibration or force-monitoring sensors have also shown that wavelength-shift responses can be used to extract frequency information for cable-force or structural dynamic assessment [37,48].

Compared with these published findings, the present study shows a similar level of frequency-domain consistency between an alternative sensing system and a conventional reference measurement. For the common frequencies identified by both PCB and FBG measurements, the overall mean relative difference was 0.94%, and the maximum relative difference was 4.26%. These values are within the range reported in previous studies that compared alternative sensing techniques with accelerometer-based or analytical reference results. This indicates that the FBG sensing system can provide reliable frequency-domain information for the dynamic characterization of stay cables. A comparison with relevant published studies is provided in Table 7 to highlight how the present PCB–FBG frequency agreement relates to previous applications of accelerometer-based, fiber-optic, and alternative sensing techniques for bridge and stay-cable dynamic characterization.

Nevertheless, the comparison also highlights an important limitation. In the present study, the FBG sensor detected several additional frequency components, especially at Locations B and D, that were not confirmed by the PCB accelerometer. Similar observations have been reported in studies using alternative sensing technologies, where additional peaks may be associated with higher sensitivity to certain response components, sensor location effects, local deformation sensitivity, or differences in the measured physical quantity.

A further limitation is that no independent temperature-compensation scheme was implemented during the field test. This limitation is less critical for the present short-term frequency-domain comparison than for long-term static strain monitoring, because the modal frequencies were identified from the dynamic components after detrending and band-pass filtering. Nevertheless, temperature compensation should be included in future long-term FBG-based stay-cable monitoring to separate thermal wavelength shifts from strain-induced responses.

## 5. Conclusions

This study presented a field-based comparative assessment of FBG sensors and PCB accelerometers for the dynamic characterization of stay cables. The comparison was conducted on selected stay cables of My Thuan Bridge under normal operating conditions. Because the two sensing systems measure different physical quantities, acceleration for the PCB accelerometer and wavelength shift associated with dynamic strain for the FBG sensor, the assessment focused on frequency-domain characteristics rather than direct time-domain amplitude equivalence.

The results show that both sensing systems provided very similar estimates of the common identified frequencies. Across all tested locations, 29 common PCB–FBG frequency components were identified. For these common components, the mean absolute frequency difference was 0.093 Hz, and the mean relative frequency difference was 0.94%. The maximum relative difference was 4.26%. These results indicate that FBG measurements can provide frequency-domain information that is generally consistent with PCB accelerometer measurements for the common detected vibration components.

The agreement between the two sensing systems was not uniform across all locations. Location C showed the strongest consistency, with the highest FBG-confirmed-by-PCB ratio and Dice similarity coefficient, while Locations B and D exhibited a larger number of FBG-only frequency components. These unmatched components may be related to differences in measured physical quantity, local sensor sensitivity, installation position, signal-to-noise level, and SSI-COV pole-selection criteria. Therefore, they should not be interpreted as independently validated physical modes based only on their detection in the FBG measurements.

The study is limited to field measurements on selected stay cables of a single bridge under normal operational excitation. Future work should include longer-term monitoring, temperature compensation, independent validation of FBG-only frequency components, and analytical or numerical modeling of the tested cables. Further studies using additional reference sensors and controlled excitation conditions would also help clarify the role of FBG sensing in identifying weak or higher-order cable vibration components.

## Figures and Tables

**Table 1:** Main specifications of the PCB accelerometer system.

Parameter	Specification
Accelerometer model	PCB 393B12
Sensor type	High-sensitivity seismic ICP^®^ accelerometer
Measured quantity	10.000 mV/g
Measurement range	0.5 g pk
Frequency range	0.15–1000 Hz, ±5%
Broadband resolution	0.000008 g rms
Data-acquisition chassis	National Instruments CompactDAQ 9178
Sampling mode	Simultaneous sampling
Maximum sampling rate of module	51.2 kS/s
Sampling frequency used in this study	2048 Hz
Acquisition duration	600 s

**Table 2:** Main specifications of the FBG sensors used in the field test.

Parameter	Value
Sensor origin	FBG Korea
Sensor model	FBG-ST-310
Center wavelength used in this study	1571.28843 nm and 1537.09553 nm
Manufacturer wavelength range	1511–1590 nm
Reflectivity	≥70%
Bandwidth/FWHM used in this study	≤0.1 nm
Manufacturer FWHM specification	≤0.3 nm
Grating length	8 mm
Measurement range	±2000 με
Strain-response specification	≥1000 με under 120 g tension
Temperature sensitivity	12 pm/°C
Manufacturer gauge-length range	100–1500 mm
Wavelength resolution	<0.5 pm
Sampling frequency	13,336.9 Hz
Measured output	Wavelength shift associated with dynamic strain

**Table 3:** Sensor installation information at the tested stay cables.

Location	Tested Cable Position	FBG Installation	PCB Accelerometer Installation	Relative Position Between PCB and FBG	Reason for Selected Installation Zone
B	Outermost stay cable in the selected cable plane	FBG sensor bonded along the cable axis with an effective bonding length of approximately 300 mm. The FBG position was represented by the midpoint of the bonded region.	PCB 393B12 accelerometer mounted on the cable surface as a point sensor within the same local measurement zone. The PCB position was represented by the center of the accelerometer mounting point.	The PCB accelerometer was installed adjacent to the FBG bonded region, close to but not exactly co-located with the FBG midpoint because of the finite bonding length required for the FBG sensor.	The location was selected away from the anchorage and damping devices to reduce local boundary effects and to obtain a representative vibration response of the free cable segment.
C	8th stay cable counted from pylon 1	FBG sensor bonded along the cable axis with an effective bonding length of approximately 300 mm. The FBG position was represented by the midpoint of the bonded region.	PCB 393B12 accelerometer mounted on the cable surface as a point sensor within the same local measurement zone. The PCB position was represented by the center of the accelerometer mounting point.	The PCB accelerometer was installed adjacent to the FBG bonded region, close to but not exactly co-located with the FBG midpoint because of the finite bonding length required for the FBG sensor.	The location was selected to represent an intermediate stay cable and to capture cable vibration behavior under normal traffic and ambient excitation.
D	Outermost stay cable in the selected cable plane	FBG sensor bonded along the cable axis with an effective bonding length of approximately 300 mm. The FBG position was represented by the midpoint of the bonded region.	PCB 393B12 accelerometer mounted on the cable surface as a point sensor within the same local measurement zone. The PCB position was represented by the center of the accelerometer mounting point.	The PCB accelerometer was installed adjacent to the FBG bonded region, close to but not exactly co-located with the FBG midpoint because of the finite bonding length required for the FBG sensor.	The location was selected away from the anchorage and damping devices to reduce local boundary effects and to obtain a representative vibration response of the free cable segment.

**Table 4:** Dominant dynamic frequencies identified from PCB accelerometer and FBG sensor.

Location	Mode/Peak	f_PCB (Hz)	f_FBG (Hz)	Absolute Difference (Hz)	Relative Difference (%)
B	1	-	2.132	-	-
	2	2.826	2.826	0	0
	3	3.51	3.521	0.011	0.31
	4	4.188	4.178	0.01	0.24
	5	-	4.837	-	-
	6	-	5.522	-	-
	7	-	6.193	-	-
	8	-	6.896	-	-
	9	9.963	9.953	0.01	0.1
	10	12.111	12.131	0.02	0.17
	11	18.094	18.124	0.03	0.17
	12	-	22.911	-	-
	13	-	23.45	-	-
C	1	-	0.221	-	-
	2	-	1.227	-	-
	3	2.231	2.231	0	0
	4	3.174	3.235	0.061	1.9
	5	4.189	4.29	0.101	2.38
	6	5.342	5.332	0.01	0.19
	7	6.375	6.155	0.22	3.51
	8	7.449	7.451	0.002	0.03
	9	8.535	8.492	0.043	0.51
	10	9.544	9.524	0.02	0.21
	11	10.729	10.529	0.2	1.88
	12	11.713	11.533	0.18	1.55
	13	12.802	12.802	0	0
	14	13.808	13.806	0.002	0.01
	15	14.95	14.82	0.13	0.87
	16	15.981	15.831	0.15	0.94
	17	17.025	16.842	0.183	1.08
	18	18.088	17.918	0.17	0.94
	19	19.091	18.923	0.168	0.88
D	1	2.943	2.911	0.032	1.09
	2	3.682	3.691	0.009	0.24
	3	4.401	4.412	0.011	0.25
	4	5.163	5.171	0.008	0.15
	5	-	5.931	-	-
	6	-	6.702	-	-
	7	-	7.462	-	-
	8	-	8.089	-	-
	9	-	8.995	-	-
	10	-	9.768	-	-
	11	-	10.545	-	-
	12	11.819	11.326	0.493	4.26
	13	12.532	12.108	0.424	3.44
	14	-	13.601	-	-
	15	-	13.946	-	-
	16	-	14.945	-	-
	17	-	17.68	-	-
	18	-	18.025	-	-
	19	-	18.762	-	-
	20	-	21.159	-	-
	21	-	21.956	-	-

**Table 5:** Summary of frequency agreement indicators.

Location	Number of PCB Peaks	Number of FBG Peaks	Number of Common PCB–FBG Peaks	FBG-Confirmed-by-PCB Ratio (%)	Dice Similarity Coefficient (%)	Mean Absolute Difference (Hz)	Maximum Absolute Difference (Hz)	Mean Relative Difference (%)	Maximum Relative Difference (%)
B	6	13	6	46.15	63.16	0.014	0.030	0.16	0.31
C	17	19	17	89.47	94.44	0.103	0.220	0.99	3.51
D	6	21	6	28.57	44.44	0.163	0.493	1.57	4.26
Overall	29	53	29	54.72	70.73	0.093	0.493	0.94	4.26

**Table 6:** Qualitative comparison of general practical characteristics of PCB accelerometers and FBG sensors based on literature-reported sensor properties and field deployment observations.

Criterion	PCB Accelerometer	FBG Sensor
Direct acceleration measurement	High	Not applicable
Frequency identification	High	High
Number of detected components	Moderate	High
Installation complexity	Moderate	Low–moderate
Multiplexing capability	Low	High
Long-term monitoring potential	Moderate	High
Sensitivity to installation quality	Moderate	High

**Table 7:** Comparison of the present results with published studies on bridge or cable dynamic monitoring.

Study	Structure/Application	Sensing Technique	Reference/Comparison Basis	Main Reported Finding	Relation to the Present Study
Gentile et al. [46]	Stay cables of cable-stayed bridges	Microwave interferometry	Conventional accelerometers	Cable natural frequencies identified from remote sensing were comparable to accelerometer-based results	Supports the use of alternative sensing systems for frequency-domain cable monitoring
Xiao et al. [47]	Long-span bridge dynamic monitoring	FBG tiltmeter	Analytical/reference dynamic results	Primary natural frequencies were identified with a maximum difference of about 5.3%	Shows that FBG-based systems can extract bridge dynamic frequencies with acceptable agreement
Zhu et al. [48]	Cable force/vibration monitoring	FBG vibration sensor	Frequency-based cable-force estimation	FBG wavelength-shift response was used to extract vibration frequency information for cable assessment	Supports the use of FBG responses for frequency-domain cable characterization
Present study	Full-scale stay cables of My Thuan Bridge	FBG sensor and PCB accelerometer	Direct PCB–FBG comparison under operational conditions	Mean relative difference of common frequencies = 0.94%; maximum relative difference = 4.26%	Provides direct field-based evidence of frequency-domain consistency between FBG and PCB measurements

## Data Availability

Data will be made available on request.
